# Up-regulation of miR-146a increases the sensitivity of non-small cell lung cancer to DDP by downregulating cyclin J

**DOI:** 10.1186/s12885-017-3132-9

**Published:** 2017-02-15

**Authors:** Lin Shi, Zhaozhong Xu, Gang Wu, Xiaoting Chen, Yuanyuan Huang, Yanjing Wang, Weiqiang Jiang, Bin Ke

**Affiliations:** 1 Department of Traditional Chinese Medicine, Zhujiang Hospital of Southern Medical University, Guangzhou, Guangdong 510282 People’s Republic of China; 2 Department of Emergency, Zhujiang Hospital of Southern Medical University, Guangzhou, Guangdong 510282 People’s Republic of China; 3 Department of Cancer Center, Zhujiang Hospital of Southern Medical University, Guangzhou, Guangdong 510282 People’s Republic of China; 4 Department of VIP & Traditional Chinese Medicine, Sun Yat-sen University Cancer Center, Guangzhou, Guangdong 510060 People’s Republic of China; 5 Department of Traditional Chinese Medicine, The First Affiliated Hospital of Sun Yet-sen University, 58 Second Zhongshan Road, Guangzhou, Guangdong 510080 People’s Republic of China

**Keywords:** miR-146a, NSCLC, DDP-resistance, CCNJ

## Abstract

**Background:**

Cisplatin (DDP)-based chemotherapy is the common first-line therapy for lung cancer. However, their efficacy is often limited by primary drug resistance and/or acquired drug resistance. The aim of this study was to investigate the function of miRNA-146a (miR-146a) in DDP-resistant non-small cell lung cancer (NSCLC), as well as the underlying mechanisms.

**Methods:**

The effect of overexpression of miR-146a and/or knockdown of cyclin J (CCNJ) in A549/DDP and SPC-A1/DDP cells were investigated as follows. The cellular sensitivity to DDP, cell apoptosis, cell cycle and cell mobility were detected by CCK-8, flow cytometry, hoechst staining and cell invasion/migration assay, respectively. The effects of miR-146a overexpression in NSCLC resistant cells were further analyzed in a nude mouse xenograft model.

**Results:**

Overexpression of miR-146a and/or knockdown of CCNJ significantly increased the sensitivity to DDP in A549/DDP and SPC-A1/DDP cells compared to NC group via arresting cell cycle, enhancing cell apoptosis, inhibiting cell viability and motility in vitro and in vivo. Furthermore, miR-146a could specially degrade the mRNA of CCNJ, as examined by dual luciferase report assay.

**Conclusion:**

The study indicates a crucial role of miR-146a in the development of acquired drug resistance to DDP in NSCLC cells. Further understanding of miR-146a mediated crosstalk networks may promote the clinical use of miR-146a analogue in NSCLC therapy.

## Background

Lung cancer is one of the most common malignant tumors and has one of the highest mortality rats worldwide [1], 80–85% of which are non-small cell lung cancer (NSCLC) [2]. The cis-diamminedichloroplatinum (II) (cisplatin, cDDP, DDP)-based chemotherapy is the common first-line therapy for clinical treatment of various malignant tumor, including lung cancer for more than 40 years [3–5]. Unfortunately, its efficacy is often limited due to the development of resistance to DDP-based therapy [2, 6]. Although more and more studies have described the resistance to DDP in NSCLC, the underlying mechanisms are not fully elucidated at present [7–10]. Therefore, a better understanding of these mechanisms of DDP resistance in NSCLC will aid the clinicians to improve NSCLC treatment and develop new targets for tumor chemoresistance.

MicroRNAs (miRs) are a superfamily of small non-coding RNAs with single-stranded 19–25 nucleotides, which could bind to the 3′-untranslated region (3′-UTR) of their targeted genes, resulting in mRNAs cleavage and/or translational repression [11, 12]. Functionally, miRs have been widely involved in the regulation of various biological processes, including embryonic development, cell cycle, differentiation, proliferation, migration, and apoptosis [13–15]. In addition, increasing studies have shown that dysregulation of miRNAs are associated with the chemoresistance in the initiation and progression of cancers [16–30]. Recently, miR-146a has been demonstrated to be up-regulated in various cancers, such as cervical cancer [31] and thyroid cancer [32, 33]. Moreover, miR-146a levels have therapeutic potential to suppress invasion and migration capacity in breast cancer [34] and pancreatic cancer [35]. However, there have been no published data regarding the roles of miR-146a in drug resistance of NSCLC cells.

In this study, we aimed to investigate the role of miR-146a in the chemosensitivity of NSCLC cells to DDP by analyzing its function in vitro and vivo. Combined with our previously data that miR-146a were significantly down-regulated in the A549/DDP cells compared with A549 cells (data was not shown), we further found that up-regulation of miR-146a markedly inhibited the migration, invasion and reversed the chemoresistance of NSCLC cells partially through targeting CCNJ. Our findings might provide a new therapeutic strategy for NSCLC patients with acquired resistance to DDP.

## Methods

### Cell lines and reagents

Human embryonic kidney 293T cells (Cat no. SCSP-502) were obtained from the Cell Bank of Chinese Academy of Science (Shanghai, China) and maintained in DMEM medium containing 10% FBS. A549 and A549/DDP cells were purchased from BioLeaf Biotech (Shanghai, People’s Republic of China). SPC-A and SPC-A1/DDP cells were obtained from Department of Molecular Biology and Biochemistry (Nanjing Medical University). A549 and SPC-A1 cells were maintained in RPMI-1640 (Hyclone, Cat no. SH30243.01B) supplemented with 10% FBS (BI, Cat no. 04-001-1A). A549/DDP and SPC-A1/DDP cells were cultured in containing 10% FBS RPMI-1640 supplemented with 1 μg/ml DDP (selleck, Cat no. S7786). All cells were cultured at 37 °C in a humidified incubator containing 5% CO_2_. To avoid the effects of the drugs, resistant cell lines were cultured in a drug-free medium for 1 week prior to further experiments.

### Construction of A549/DDP and SPC-A1/DDP stable cells clones with miR-146a overexpression

MiR-146a (the full-length pri-miR-146a) were cloned into lenti-miR overexpression plasmid PGC-Lv (Genechem, Cat no. GV235). MiR-146a and control (NC) plasmid were packaged with lenti-packaging plasmid mix (pHelper 1.0, and pHelper 2.0) in a 293T packaging cell line. Viruses were concentrated and purified using ultracentrifugation. Transfection was performed with Lipofectamine 2000 (Invitrogen, Carlsbad, CA) according to the manufacturer’s instruction. Stably cells clones were selected with puromycin (1 μg/ml) 48 h after lentiviruses transfection, and individual clone was isolated and maintained in a medium containing puromycin (0.5 μg/ml). The expression of miR-146a was confirmed by real-time quantitative PCR (RT-qPCR).

### siRNA and transfection

A small interfering RNA (siRNA) targeting CCNJ (siCCNJ) with the sequences (sense 5′-UGGAUUUGUACCAUUCUUCUGdtdt-3′ and anti-sense 5′-GAAGAAUGGUACAAAUCCAAGdtdt-3′) and non-targeting siRNA (NC) were purchased from RiboBio (Guangzhou, China). For cell transfection, A549/DDP and SPC-A1/DDP cells were transfected with siCCNJ or NC at a final concentration of 50 nM using Lipofectamine 2000 (Invitrogen, Carlsbad, CA) following the manufacturer’s instructions. Silencing efficiency of CCNJ was determined at mRNA and protein levels by RT-qPCR and Western blotting, respectively.

### CCK-8 assay

The transfected cells were seeded into 96-well culture plates at a density of 5000 cells per well. After culture for 12 h, cells were added serially diluted DDP (0, 2, 4, 6, 8 μg/ml) and incubated for 48 h. A total of 10 μl CCK-8 solution was added to each well after treatment, followed by another 1–2 h incubation. Optical density value at 450 nm (OD_450_) was detected using the New Epoch™ 2 Epoch Microplate Spectrophotometer (Biotek, CA, USA). The IC_50_ value was calculated by nonlinear regression analysis with GraphPad Prism 5.0 (GraphPad Software Inc., San Diego, CA, USA), using the dose-response with variable slope function.

### RT-qPCR assay

Total cellular or tissular RNA was extracted using TRIzol^®^ reagent (Invitrogen, Cat no.15596-018). Approximately 1 μg of extracted total RNA sample was reverse transcribed into cDNA using PrimeScript™ RT reagent Kit (Takara, Otsu, Japan) following the manufacturer’s protocol. Quantification of miRNA or mRNA was performed using Bestar™ qPCR Master Mix (SYBR Green) according to the manufacturer’s instructions. RT-qPCR primers used were as follow: miR-146a sense, 5′-GCGAGGTCAAGTCACTAGTGGT-3′ and antisense, 5′-CGAGAAGCTTGCATCACCAGAGAACG-3′; U6 sense, 5′-CTCGCTTCGGCAGCACA-3′ and antisense, 5′-AACGCTTCACGAATTTGCGT-3′; CCNJ sense, 5′-TGTCCGTCAGAACCCATGC-3′ and antisense, 5′-AAAGTCGAAGTTCCATCGCTC-3′; GAPDH sense, 5′-GCACCGTCAAGGCTGAGAAC-3′ and antisense, 5′-TGGTGAAGACGCCAGTGGA-3′. The cycling conditions were as follow: initial denaturation at 95 °C for 60 s, 95 °C for 5 s, 58 °C for 20 s, 40 cycles. Data analysis was performed using the 2^-ΔΔCt^ method.

### Western blot

The cells were harvested and lysed using the mammalian protein extraction reagent RIPA (Beyotime, Beijing, China). Total protein concentration of the lysate was measured by BCA Protein Assay Kit (Pierce Biotechnology, Cat no. 23235). Approximately 50 μg proteins extraction in each lane were separated on 10% SDS-PAGE and transferred onto a PVDF membrane (BioRad, Cat no.162-0177). The membrane was incubated with primary antibodies overnight at 4 °C, followed by incubation of horseradish-peroxidase (HRP) conjugated goat anti-rabbit IgG antibody (Santa Cruz, Cat no. SC-2054; 1:5000 dilution). Signals intensity were measured by the ECL-PLUS/Kit (Amersham, Cat no. RPN2132) following the manufacturer’s protocol. The blots were quantified by densitometry using Quantity One software (Bio-Rad, CA, USA). GAPDH antibody was used as an internal control. The primary antibodies were as follow: rabbit anti-CCNJ (Cat. no. ab138561, 1:1000 dilution), P-gp (Cat. no. ab103477, 1:1000 dilution), MRP1 (Cat. no. ab99531, 1:1000 dilution), MVP/LRP (Cat. no. ab97311, 1:1000 dilution), P53 (Cat. no. ab31333, 1:1000 dilution), cleaved caspase-3 (Cat. no. ab2302, 1:200 dilution) were provided by Abcam (MA, USA).

### Cell cycle and apoptosis using flow cytometry

For cell cycle analysis, cells were harvested, washed with ice-cold PBS and then fixed with 70% ethanol (v/v) overnight at −20 °C. Fixed cells were washed with ice-cold PBS twice and then resuspended in PBS containing PI (50 μg/ml)/RNase A (50 μg/ml) for 10 min. For cell apoptosis analysis, cells were double stained with Annexin V-FITC (1 μg/ml) and PI (1 μg/ml). Finally, both cell cycle and apoptosis analyzed using a FACScan instrument (Becton Dickinson, Mountain view, CA, US) equipped with CellQuest software (Becton Dickinson, Mountain view, CA, US).

### Hoechst staining assay

Cells were cultured in six-well plates for 48 h after transfection and added Hoechst 33342 (1 μg/ml, Sigma, USA) for 10 min. After washed with PBS, the changes in nuclear morphology were observed with fluorescence microscopy.

### In vitro transwell assays

The cell motility was assessed by transwell migration chambers (8.0 μm pore size; 6.5 mm diameter, Corning) following manufacture’s instruction. For cell migration, approximately 5 × 10^4^ transfected cells were seeded into upper chambers in 600 μl serum free medium. For cell invasion assay, 0.2.mg/ml Matrigel^TM^ Basement Membrane Matrix (BD, Cat. no. 356234) was coated the upper chamber. The Matrigel was allowed to solidify at 37 °C overnight. After solidification, 5 × 10^4^ transfected cells in serum-free medium were seeded into the upper chamber. The lower transwell chamber contained 10% FBS RPMI-1640 was used as achemoattractant. After cultured for 24 h, the cells were removed from the upper surface and then fixed in 4% paraformaldehyde and finally stained with 0.1% crystal violet solution. For quantification, the migratory cells and invasion cells were counted under a microscope in five random fields. Each experiment was carried out in triplicate and the mean values were presented.

### Dual-luciferase reporter assay

A CCNJ-WT (wild type of miR-146a binding site in 3′-UTR of CCNJ) and CCNJ-MUT (miR-146a binding site in 3′-UTR of CCNJ) luciferase reporter was constructed by Genechem (Shanghai, China). Briefly, the wild type and mutated of 3′-UTR sequence of CCNJ predicted to interact with miR-146a were synthesized by Genechem, and cloned into psi-CHECK2 vector (Promega, Madison, WI, USA). The constructs were sequenced and named as CCNJ/3′-UTR-WT or CCNJ/3′-UTR-MUT. For reporter assays, 0.05 μg firefly luciferase reporter plasmid psi-CHECK2 and 0.01 μg renilla luciferase (internal reference vector) was co-transfected into 293T cells which transfected with lentivirus containing miR-146a or NC using Lipofectamine 2000 in 96-well plates. Luciferase activity (fluorescence intensity) was determined using fluorophotometer 36 h after transfection.

### Xenograft transplantation and in vivo chemosensitivity assay

A549 cells and A549/DDP cells transfected with miR-146a or NC (5 × 10^6^ in 0.2 ml of PBS) were suspended in Matrigel mixture and were subcutaneously injected into BALB/c nude mice (Nu/Nu, female, 4–6 weeks old, *n* = 8/group), which were purchased from Sun Yat-sen University (Guangdong, China) and maintained under pathogen-free conditions. After one week, mice were treated with DDP (3.0 mg/kg body weight; per 3 days). Tumor volume was monitored for 4 weeks and measured per 3 or 4 days. The tumor volume formed was calculated by the following formula: Volume = (Length × width^2^) × 0.5. Then mice were euthanized by cervical dislocation. Tumors were harvested and divided into two parts: half of each tumor was frozen in liquid nitrogen and stored at −80 °C for RT-qPCR and Western blotting analysis. The other half was fixed in 4% paraformaldehyde for TUNEL assay, H&E and IHC analysis as previously described [30, 36].

### Bioinformatic and statistical analysis

Online miRNA databases (miRBase, TargetScan, MiRanda, and PicTarget) were used to predict the target gene of miR-146a. The Graph pad prism 5.01 software system was used for statistical analysis. Data are expressed as the mean ± SD. All experiments were repeated three times. The statistical significance of the results between each group was evaluated using one-way ANOVA or *t*-test. Differences were considered significant at *p < 0.05, **p < 0.01, and ***p < 0.001.

## Results

### Overexpression of miR-146a enhanced the sensitivity ofA549/DDP and SPC-A1/DDP cells to DDP

**Fig. 1:**
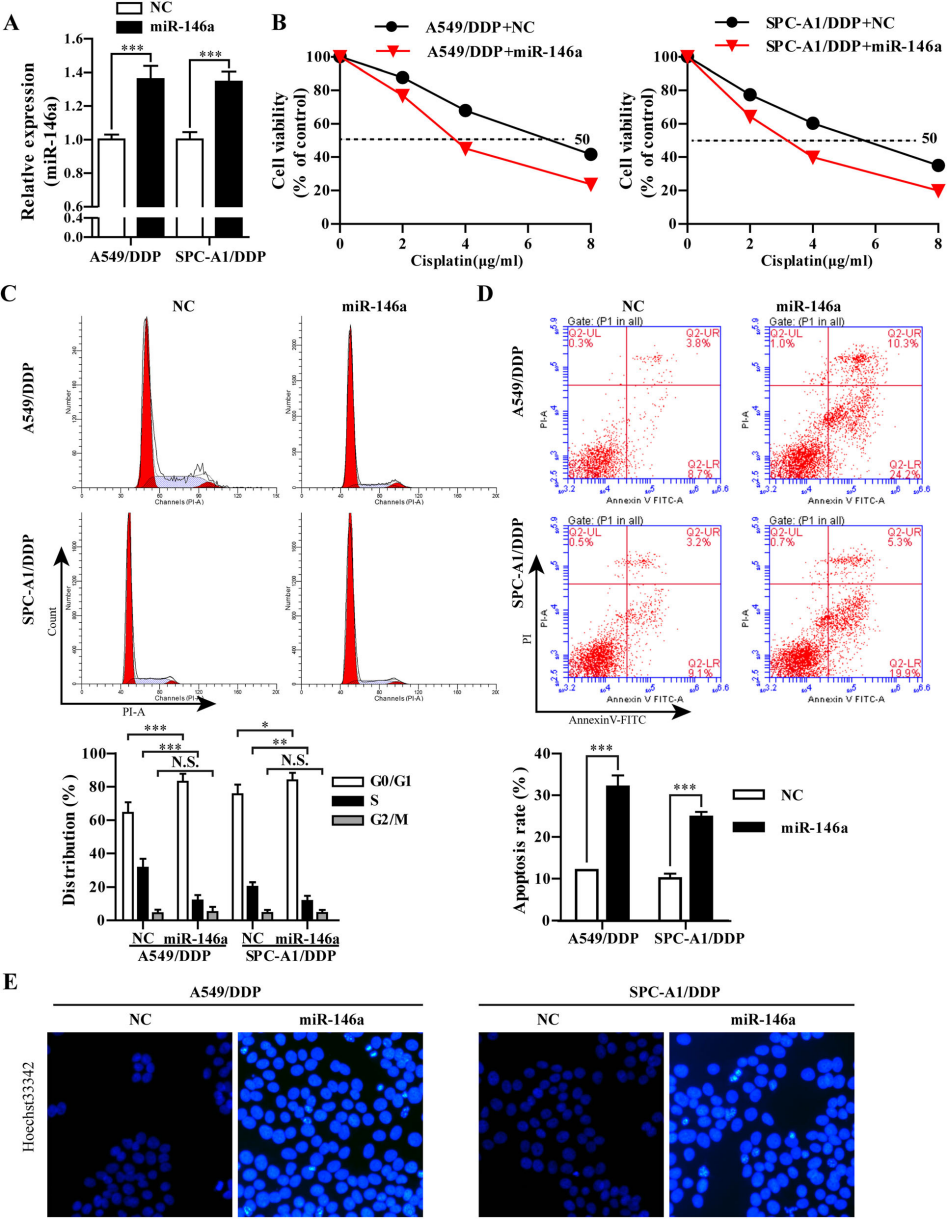
Overexpression of miR-146a enhanced the sensitivity to DDP in A549/DDP and SPC-A1/DDP cells. **a** The mRNA levels of miR-146a in A549/DDP and SPC-A1/DDP cells stably transfected with miR-146a or NC lentivirus were analyzed by RT-qPCR. **b** The cell vitality was evaluated by CCK-8 assay, A549/DDP and SPC-A1/DDP cells stably transfected with miR-146a or NC lentivirus treated with serially diluted DDP. **c** Representative data of FACS statistical graph analyses of cell cycle in A549/DDP and SPC-A1/DDP cells stably transfected with miR-146a or NC lentivirus and then treated with 1 μg/ml DDP for 48 h. **d** Representative data of FACS and statistical graph analyses of cell apoptosis in A549/DDP and SPC-A1/DDP cells stably transfected with miR-146a or NC lentivirus and incubated with 1 μg/ml DDP for 48 h. **e** Representative data of Hoechst staining assay in A549/DDP and SPC-A1/DDP cells stably transfected with miR-146a or NC lentivirus and then treated with 1 μg/ml DDP for 48 h. All data were expressed as mean value ± SD from 3 independent experiments. N.S. = no significant, *p < 0.05, **p < 0.01, ***p < 0.001

**Fig. 2:**
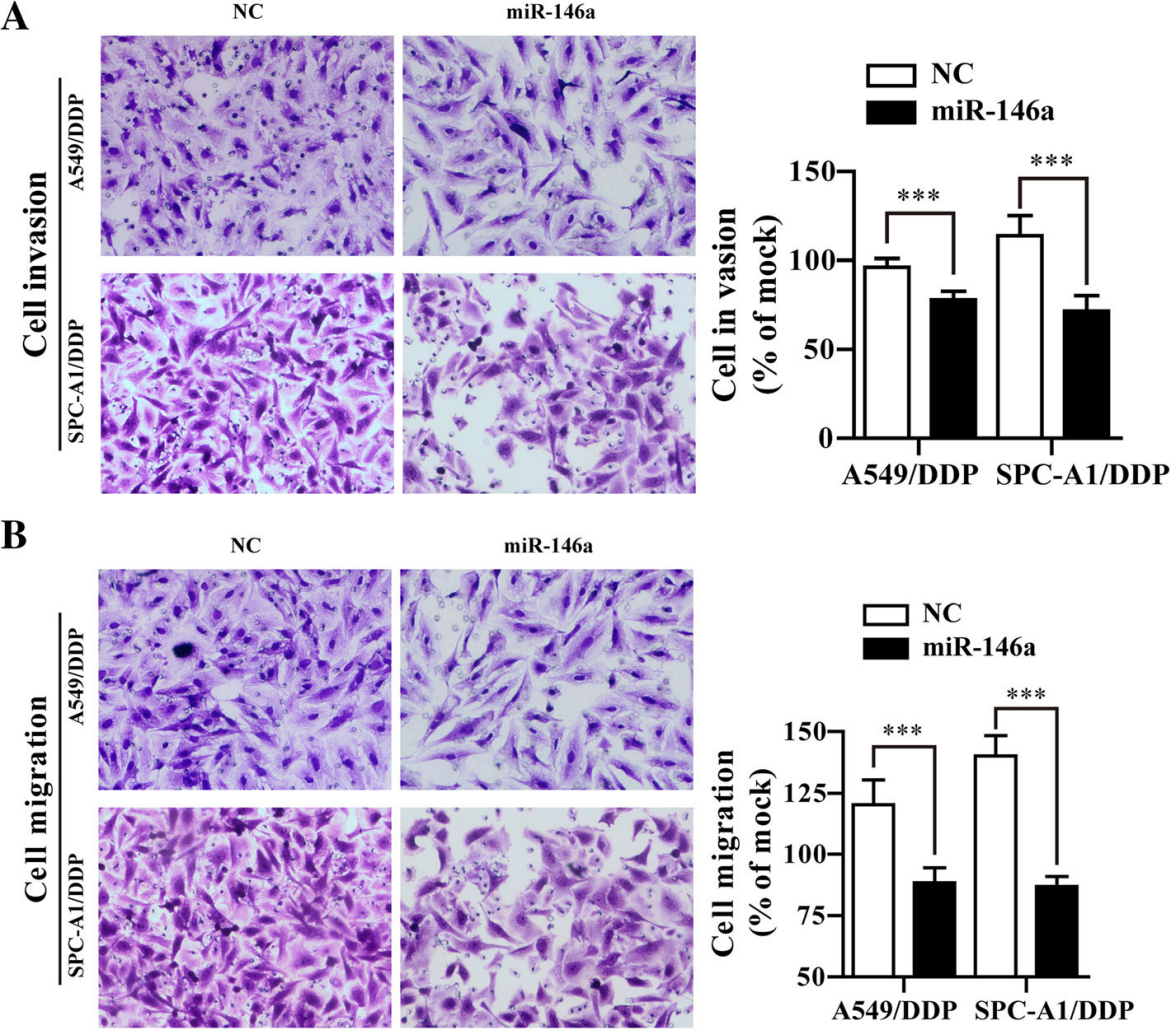
Overexpression of miR-146a inhibited the cell invasion and migration in A549/DDP and SPC-A1/DDP cells. **a** Representative data of cell invasion assay and statistical graph in A549/DDP and SPC-A1/DDP cells stably transfected with miR-146a or NC lentivirus and treated with 1 μg/ml DDP for 24 h. **b** Representative data of cell migration assay and statistical graph in A549/DDP and SPC-A1/DDP cells stably transfected with miR-146a or NC lentivirus and incubated with 1 μg/ml DDP for 24 h. All data were expressed as mean value ± SD from 3 independent experiments. ***p < 0.001

Our previously micro-array data have shown that miR-146a were down-regulated in the A549/DDP cell compared with A549 cell (data was not shown). To deeply investigate the roles of miR-146a in the DDP resistance in DDP-resistant NSCLC cells, the stably overexpressed miR-146a cell lines were established by transfecting miR-146a into A549/DDP and SPC-A1/DDP cells, respectively. As shown in Fig. 1a, miR-146a expression was significantly increased in A549/DDP/miR-146a and SPC-A1/DDP/miR-146a cells compared with A549/DDP/NC and SPC-A1/DDP/NC. CCK-8 assay was then performed to determine the effects of miR-146a on cell viability of A549/DDP and SPC-A1/DDP cells when exposed to DDP treatment. The IC_50_ values for DDP were decreased from 7.69 ± 0.733 μg/ml in A549/DDP/NC to 3.76 ± 0.388 μg/ml in A549/DDP/miR-146a cells. Similarly, overexpression of miR-146a significantly reduced the IC50 values from 5.48 ± 0.641 μg/ml to 2.41 ± 0.339 μg/ml in SPC-A1 cells (Fig. 1b), which indicated that overexpression of miR-146a could partially reverse the DDP-resistant NSCLC cells. Next, we analyzed the effects of miR-146a on cell cycle and apoptosis of A549/DDP and SPC-A1/DDP cells by flow cytometry. Compared with A549/DDP/NC and SPC-A1/DDP/NC cells, the percentage of G0/G1 phase was increased and S phase was decreased in both A549/DDP/miR-146a and SPC-A1/DDP/miR-146a cells under 1 μg/ml DDP treatment (Fig. 1c). Also, miR-146a could significantly increase apoptosis rate of A549/DDP (34.5 vs 12.5%) or SPC-A1/DDP (25.2 vs12.3%) cells compared with control group under 1 μg/ml DDP treatment (Fig. 1d, *p* < 0.001). Consistantly, hochest 33342 staining revealed obviously decrease in the nuclei of live cells (blue color) (Fig. 1e). Collectively, up-regulation of miR-146a could reverse the chemo-resistance to DDP in NSCLC cells by inducing cell-cycle arrest in G0/G1 phase and apoptosis. In addition, we assessed cell invasion and migration through a transwell assay. As shown in Fig. 2a and b, overexpression of miR-146a significantly impaired the invasion and migration ability of A549/DDP or SPC-A1/DDP cell under 1 μg/ml DDP treatment (*p* < 0.001).

### CCNJ was identified as a functional target of miR-146a

**Fig. 3:**
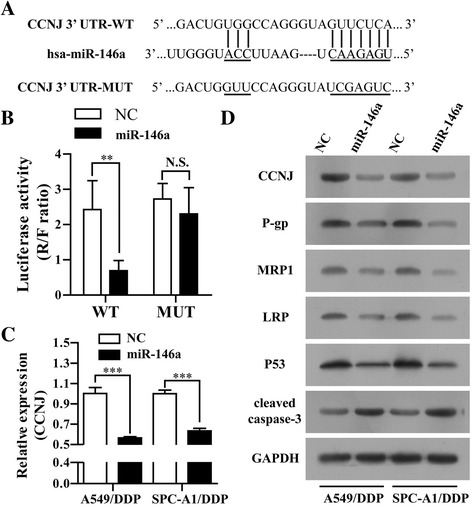
CCNJ was a novel target gene of miR-146a. **a** The binding site of miR-146a and the prediction target genes (CCNJ) through Targetscan web. **b** The wild type (CCNJ 3′-UTR-WT) or mutant (CCNJ 3′-UTR-MUT) reporter plasmids were co-transfected into 293T cells with miR-146a or NC lentivirus. The normalized luciferase activity in the control group was set as relative luciferase activity. **c** The mRNA levels of CCNJ in A549/DDP and SPC-A1/DDP cells stably transfected with miR-146a or NC lentivirus and incubated with 1 μg/ml DDP for 48 h were analyzed by RT-qPCR. **d** The protein levels of CCNJ, P-gp, MRP1, LRP, P53 and cleaved Caspase-3 were analyzed in A549/DDP and SPC-A1/DDP cells stably transfected with miR-146a or NC lentivirus by Western blot. All data represented as mean value ± SD from 3 independent experiments. N.S. = no significant, **p < 0.01, ***p < 0.05

As we know, miRs exert their function by affecting their target genes expression. Thus, the target genes of miR-146a were predicted through four publicly available web (miRBase, TargetScan, MiRanda, and PicTarget), and CCNJ was selected as a putative target (Fig. 3a). To directly address whether miR-146a binds to the 3′-UTR of CCNJ mRNA, we constructed luciferase reporters carrying the 3′-UTR with the putative miR-146a binding sites for CCNJ mRNA. Correspondingly, we also generated a mutant reporter vector which contains the CCNJ3′-UTR with a mutation at the putative miR-146a binding site. Dual-luciferase reporter assay showed that miR-146a significantly inhibited the relative luciferase activity of the report vector which contains the 3′-UTR of CCNJ construct. In contrast, no change of luciferase activity was observed in cells transfected with the mutant 3′-UTR of CCNJ constructs (Figure3B). It suggests that miR-146a might have a target site in the 3′-UTR of CCNJ mRNA. We next determined whether overexpression of miR-146a could downregulate CCNJ expression. As shown in Fig. 3c and d, the expression of CCNJ (both mRNA and protein level) was significantly lower in miR-146a overexpressed cells than that in NC group by RT-qPCR and Western blot. These results further suggest that CCNJ is a direct target of miR-146a in NSCLC cells, and miR-146a may negatively regulate the expression of CCNJ. Furthermore, we found that drug-resistance-associated proteins (P-gp, MRP1 and LRP) and P53 were decreased but apoptosis-related protein (cleaved caspase-3) was increased in A549/DDP/miR-146a and SPC-A1/DDP/miR-146a cells compared swith A549/DDP/NC and SPC-A1/DDP/NC (Fig. 3d).

### Knockdown of CCNJ solely increased the sensitivity of A549/DDP and SPC-A1/DDP cells to DDP

**Fig. 4:**
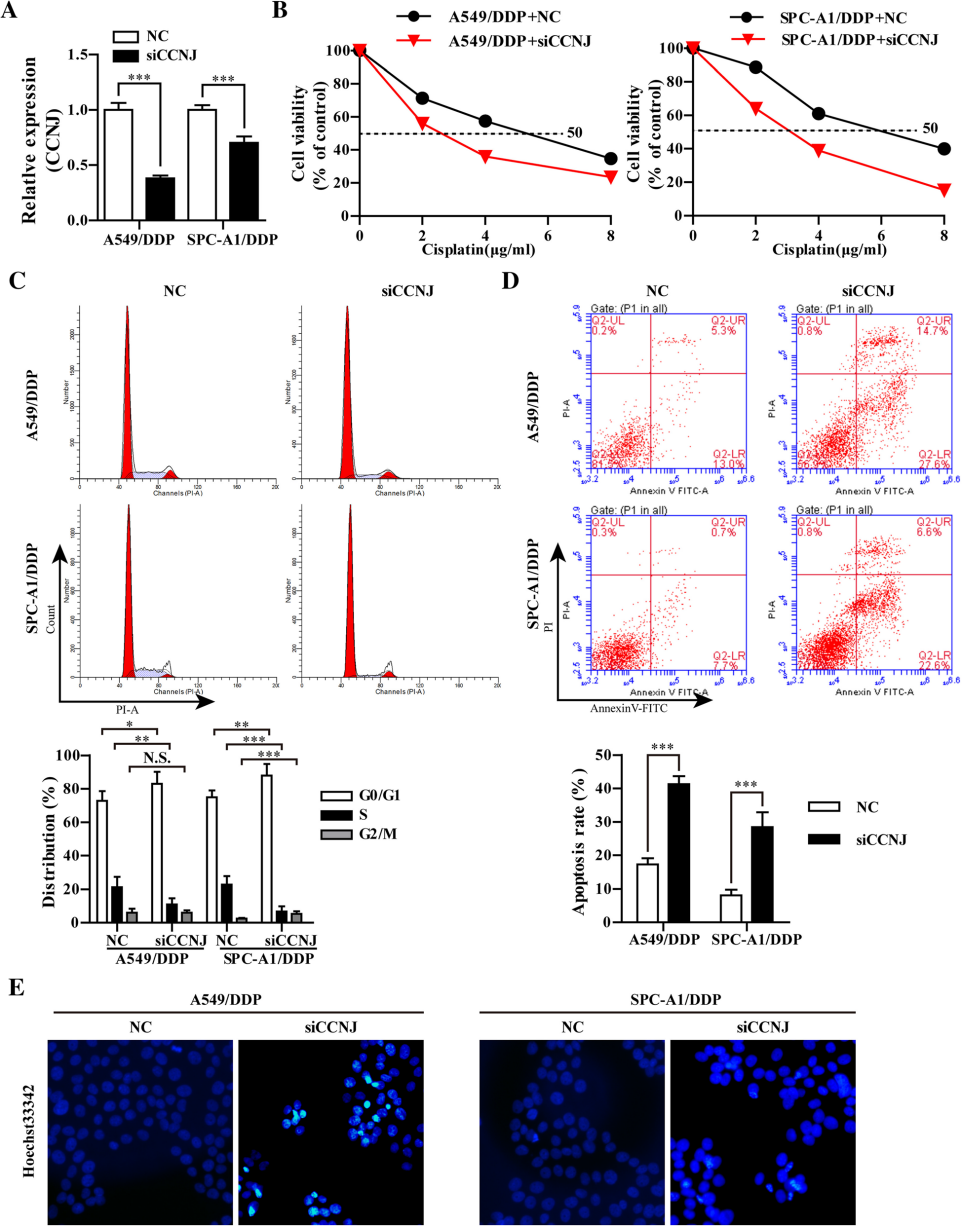
Knockdown of CCNJ enhanced the sensitivity to DDP in A549/DDP and SPC-A1/DDP cells. **a** The mRNA levels of CCNJ in A549/DDP and SPC-A1/DDP cells transfected with siCCNJ or NC were analyzed by qRT-PCR. **b** The cell viability was evaluated by CCK-8 assay, A549/DDP and SPC-A1/DDP cells transfected with siCCNJ or NC treated with serially diluted DDP. **c** Representative data of FACS statistical graph analyses of cell cycle in A549/DDP and SPC-A1/DDP cells transfect with siCCNJ or NC for 6 h and then incubated with 1 μg/ml DDP for 48 h. **d** Representative data of FACS and statistical graph analyses of cell apoptosis in A549/DDP and SPC-A1/DDP cells transfected with siCCNJ or NC for 6 h and then incubated with 1 μg/ml DDP for 48 h. **e** Representative data of Hoechst staining assay in A549/DDP and SPC-A1/DDP cells transfected with siCCNJ or NC for 6 hand then incubated with 1 μg/ml DDP for 48 h. All data represented as mean value ± SD from 3 independent experiments. N.S. = no significant, * *p* < 0.05, **p < 0.01, ***p < 0.001

**Fig. 5:**
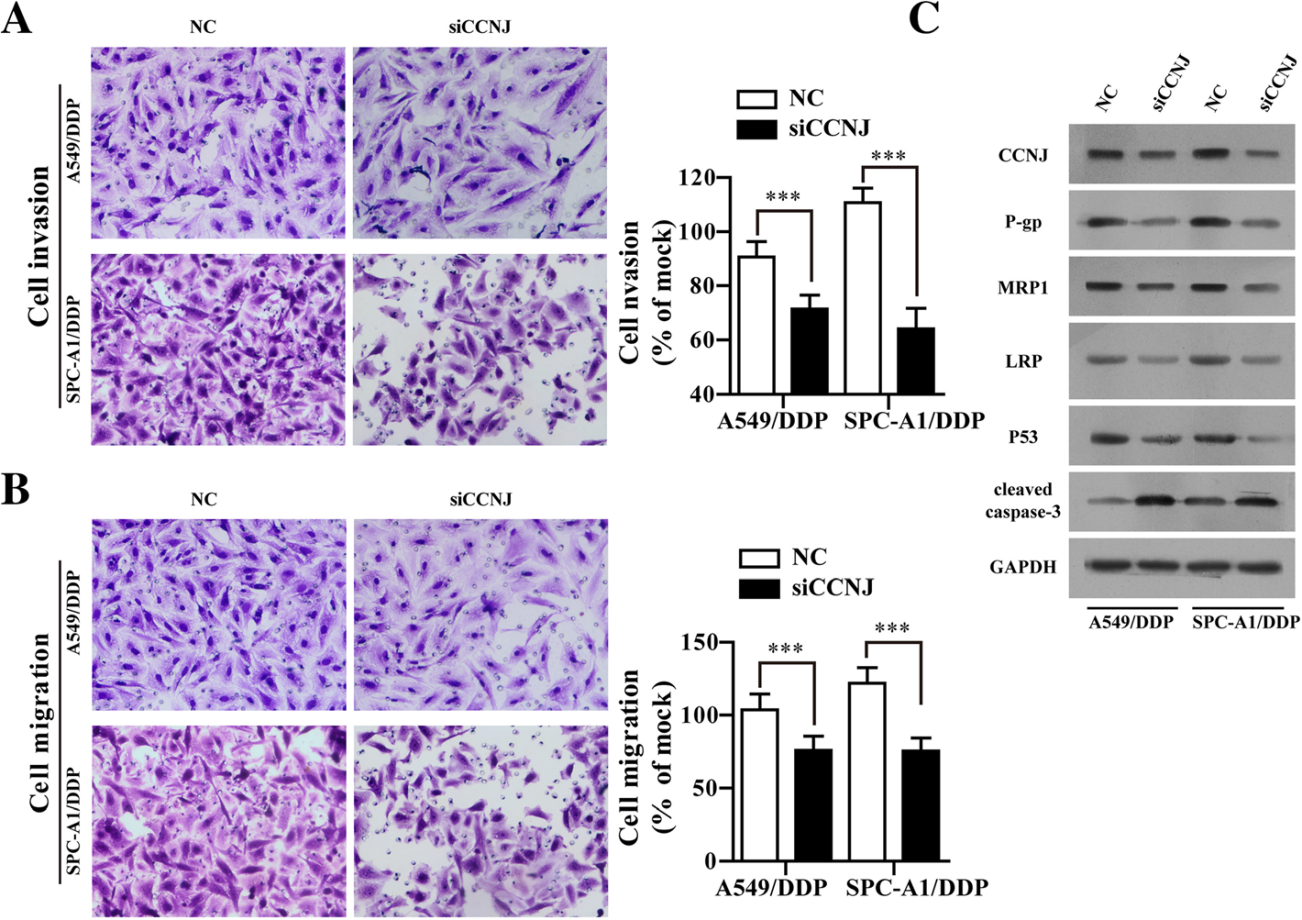
Knockdown of CCNJ inhibited the cell invasion and migration in A549/DDP and SPC-A1/DDP cells. **a** Representative data of cell invasion assay and statistical graph in A549/DDP and SPC-A1/DDP cells transfected with siCCNJ or NCfor 6 h and then incubated with 1 μg/ml DDP for 24 h. **b** Representative data of cell migration assay and statistical graph in A549/DDP and SPC-A1/DDP cells transfected with siCCNJ or NC for 6 h and then incubated with 1 μg/ml DDP for 24 h. **c** The protein levels of CCNJ, P-gp, MRP1, LRP, P53 and cleaved Caspase-3were analyzed in A549/DDP and SPC-A1/DDP cells transfected with siCCNJ or NC for 6 h and then incubated with 1 μg/ml DDP for 48 h by Western blot. All data were expressed as mean value ± SD from 3 independent experiments. ***p < 0.001

To explore the individual effect of CCNJ as a novel target gene of miR-146aon NSCLC DDP resistance, the expression of CCNJ was down-regulated using RNAi, as evidenced by RT-qPCR and Western blot assay. As expected, the mRNA and protein level of CCNJ was significantly decreased in A549/DDP/siCCNJ and SPC-A1/DDP/siCCNJ cells compared with A549/DDP/NC and SPC-A1/DDP/NC (Fig. 4a). CCK-8 assay was then performed to further assess the role of CCNJ in regulating growth of A549/DDP and SPC-A1/DPP cells exposed to DDP. As shown in Fig. 4b, IC_50_ values of A549/DDP and SPC-A1/DDP cells transfected with siCCNJ to DDP were decreased compared with that transfected with NC (4.85 ± 0.627 μg/ml vs 2.414 ± 0.339 μg/ml and 5.48 ± 0.6409 μg/ml vs 2.80 ± 0.334 μg/ml, respectively (*p* < 0.001). Under 1 μg/ml DDP treatment, knockdown of CCNJ significantly increased the percentage of A549/DDP SPC-A1/DDP cells in G0/G1 phase compared with negative controls (Fig. 4c). In cell apoptosis, the cell apoptosis of A549/DDP and SPC-A1/DDP cells which transfected with siCCNJ for 6hand then treat with 1 μg/ml DDP for 48 h were detected using Annexin V-FITC/PI flow cytometry (Fig. 4d) and Hoechst staining assay (Fig. 4e). It was observed that knockdown of CCNJ promoted the cell apoptosis of A549/DDP and SPC-A1/DDP cells (42.3 vs 18.3% and 29.2 vs 8.7%, respectively compared with controls. Therefore, knockdown of CCNJ could also reverse the DDP resistance of resistant NSCLC cells by inducing cell-cycle arrest in G0/G1 phase and enhancing apoptosis. In addition, transwell cell migration and Matrigel invasion assay was performed to examine the effect of knockdown of CCNJ on cell motility ability. The data indicated that knockdown of siCCNJ markedly inhibited the invasion and migration of NSCLC resistance cells (Fig. 5a and b). We also found that the classical drug-resistance-associated proteins (P-gp, MRP1 and LRP) and P53 were remarkably inhibited and apoptosis-related protein (cleaved caspase-3) was significantly increased in A549/DDP/siCCNJ and SPC-A1/DDP/siCCNJ cells compared with A549/DDP/NC and SPC-A1/DDP/NC (Fig. 5c). These data further indicated that CCNJ might serve as a new target gene for enhancing the chemosensitivity of NSCLC to DDP.

### CCNJ was involved in miR-146a induced sensitivity to DDP in A549/DDP and SPC-A1/DDP cells

**Fig. 6:**
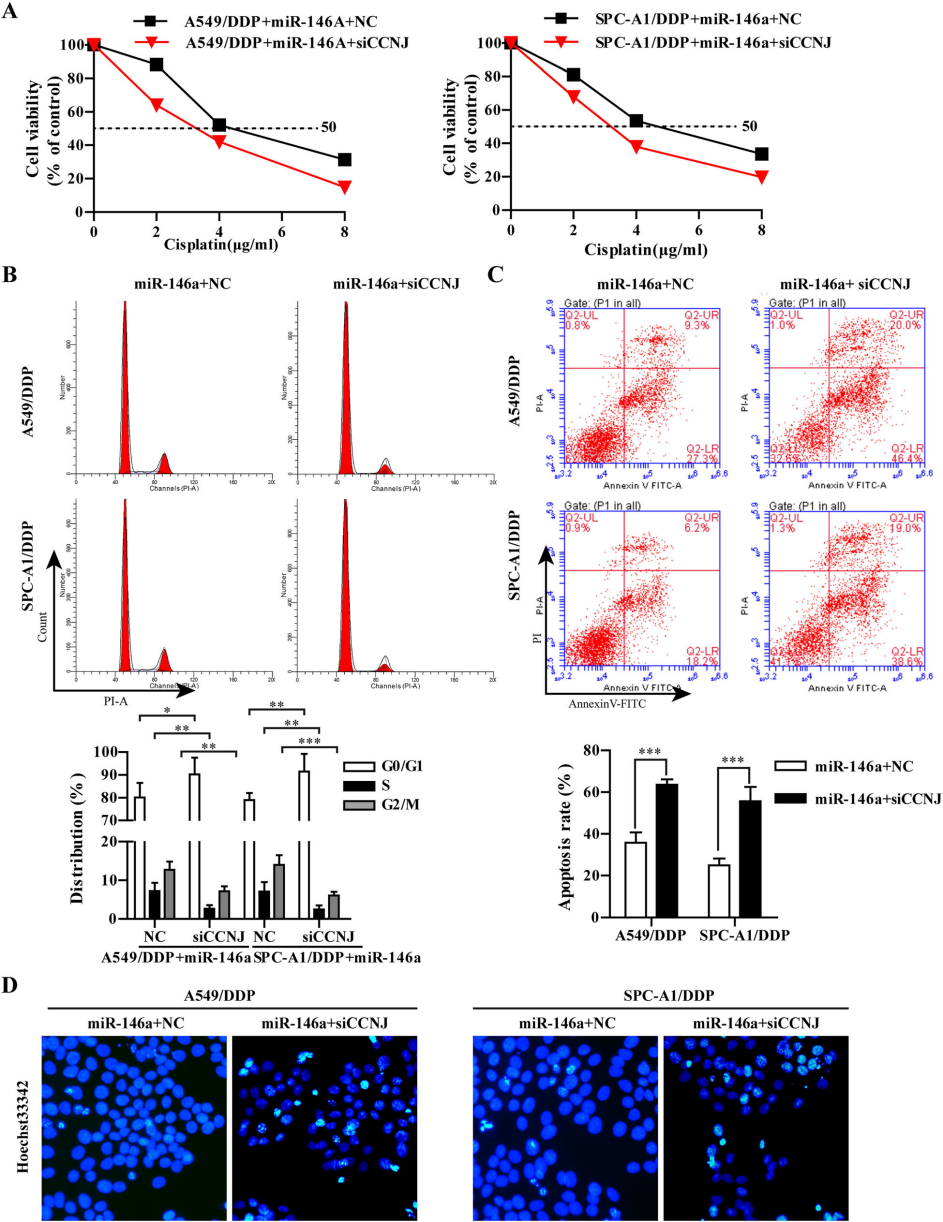
Overexpression of miR-146a and knockdown of CCNJ further enhanced the sensitivity to DDP in A549/DDP and SPC-A1/DDP cells. **a** The cell vitality was evaluated by CCK-8 assay, A549/DDP/miR-146a and SPC-A1/DDP/miR-146a cells transfected with siCCNJ or NC for 6 h and then treated with serially diluted DDP. **b** Representative data of FACS statistical graph analyses of cell cycle in A549/DDP/miR-146a and SPC-A1/DDP/miR-146a cells transfected with siCCNJ or NC for 6 h and then incubated with 1 μg/ml DDP for 48 h. **c** Representative data of FACS and statistical graph analyses of cell apoptosis in A549/DDP/miR-146a and SPC-A1/DDP/miR-146a cells transfected with siCCNJ or NC for 6 h and then incubated with 1 μg/ml DDP for 48 h. **d** Representative data of Hoechst staining assay inA549/DDP/miR-146a and SPC-A1/DDP/miR-146a cells transfected with siCCNJ or NC for 6 h and then incubated with 1 μg/ml DDP for 48 h. All data were expressed as mean value ± SD from 3 independent experiments. *p < 0.05, **p < 0.01, ***p < 0.001

**Fig. 7:**
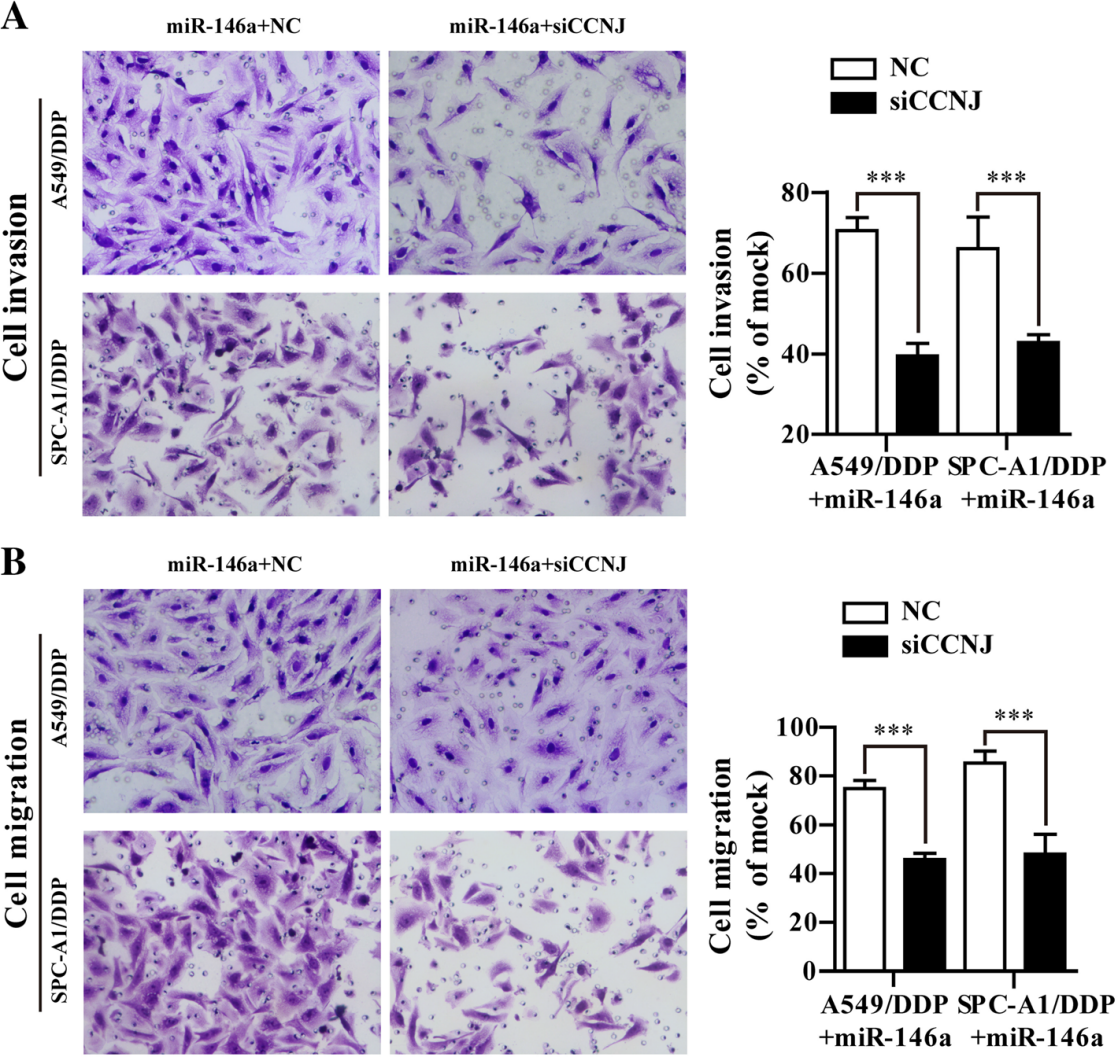
CCNJ was involved in miR-146a-induced sensitivity to DDP in A549/DDP and SPC-A1/DDP cells. **a** Representative data of cell invasion assay and statistical graph in A549/DDP/miR-146a and SPC-A1/DDP/miR-146a cells transfected with siCCNJ or NC for 6 h and then incubated with 1 μg/ml DDP for 24 h. **b** Representative data of cell migration assay and statistical graph in A549/DDP/miR-146a and SPC-A1/DDP/miR-146a cells transfected with siCCNJ or NC for 6 h and then incubated with 1 μg/ml DDP for 24 h. All data represented as mean value ± SD from 3 independent experiments. ***p < 0.001

To investigate whether CCNJ is involved in miR-146a induced chemoresistance, we performed gain-of-function and loss-of-function assays in A549/DDP and SPC-A1/DDP cells. As shown in Fig. 6a, knockdown of CCNJ significantly increased the sensitivity to DDP by 2-fold in A549/DDP/miR-146a (2.71 ± 0.273 μg/ml vs 5.25 ± 0.488 μg/ml) and SPC-A1/DPP/miR-146a cells (2.861 ± 0.3054 μg/ml vs 5.087 ± 0.5673 μg/ml, respectively compared with NC group. Cell cycle analysis was performed to determine whether there was any cell cycle alteration in NSCLC cells/DDP/miR-146a after knockdown of CCNJ. Compared with that of NC-transfected cells, the percentage of siCCNJ-transfected AA549/DDP/miR-146a and SPC-A1/DDP/miR-146a in G0/G1 phase of cell cycle increased from 83.00 to 90.18% and 79.56 to 91.43%, respectively (Fig. 6b). Furthermore, knockdown of CCNJ could significantly increase cell apoptosis (63.6% vs 38.3% in A549/DDP cells and 55.7% vs 24.8% in SPC-A1/DDP cells) compared with individual overexpression of miR-146a or knockdown of CCNJ (Fig. 6c and d). In addition, we assessed NSCLC cell invasion and migration through a transwell assay. As shown in Fig. 7a and b, combined with knockdown of CCNJ and miR-146a remarkably suppressed the invasion and migration ability induced in NSCLC resistance cells compared with sole overexpression of miR-146a or knockdown of CCNJ (*p* < 0.001). There results strongly support that knockdown of CCNJ could enhance the gains of the sensitivity to DDP in miR-146a-overexpressing A549/DDP and SPC-A1/DDP cells.

### Overexpression of miR-146a enhanced the in vivo sensitivity of A549/DDP cells to DDP

**Fig. 8:**
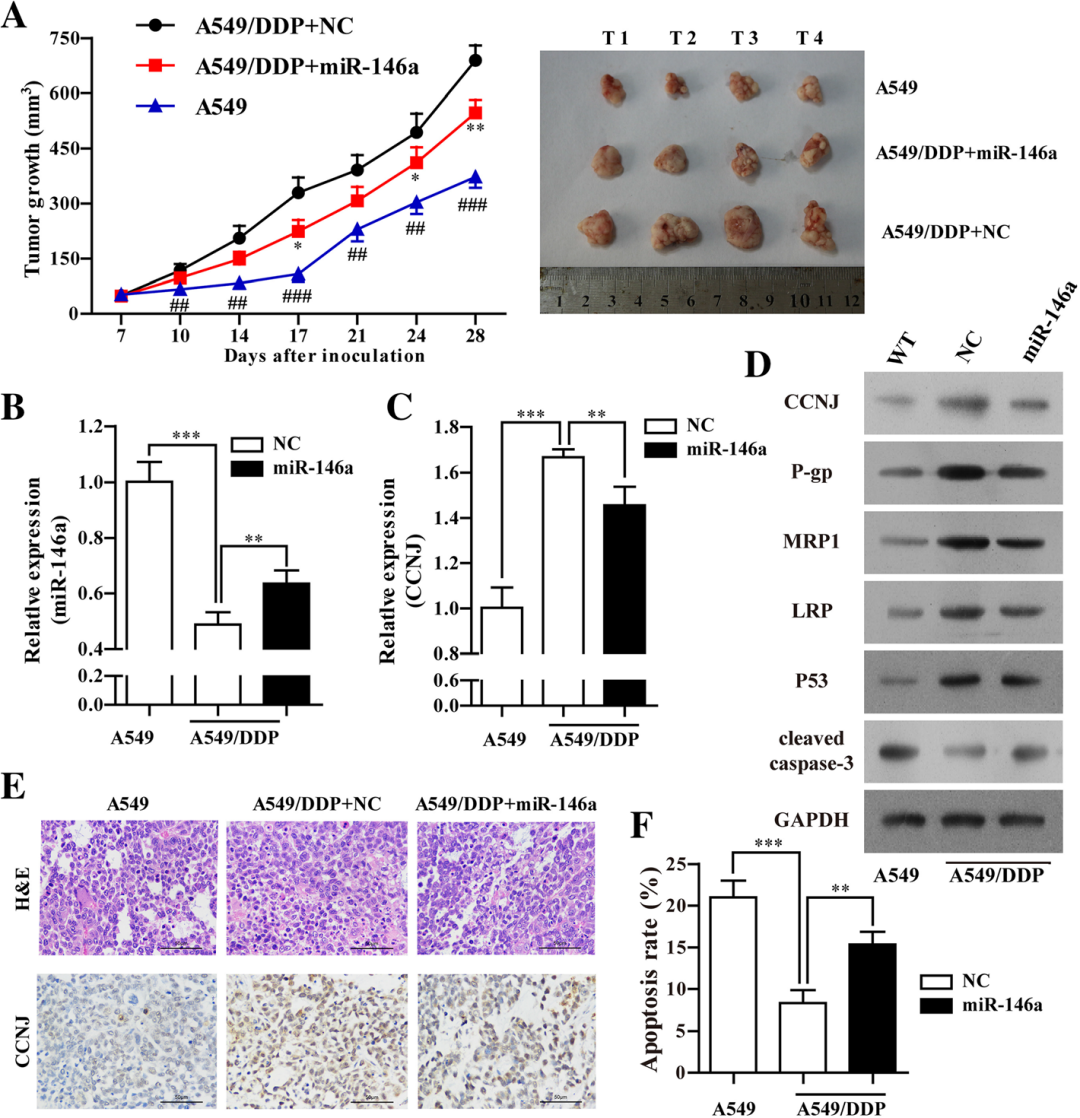
Overexpression of miR-146a enhanced the sensitivity to DDP in A549/DDP cells in vivo. **a** Growth curve of tumor volumes and tumor images of A549, A549/DDP/NC and A549/DDP/miR-146a. Each data point represents the mean ± SD of 8 mice. Quantification of miR-146a (**b**) and CCNJ mRNA levels (**c**) in the transplanted tumor tissues among A549, A549/DDP/NC and A549/DDP/miR-146a by RT-PCR; (**d**) The quantification of CCNJ, P-gp, MRP1, LRP, P53 and cleaved caspase-3 in A549, A549/DDP/NC and A549/DDP/miR-146a forming tumor by Western blot. **e** H&E (upper) and CCNJ (lower) stained sections of the transplanted tumors by IHC, magnification, 40×, Bars = 50 μm. **f** The quantification of apoptotic cell among A549, A549/DDP/NC and A549/DDP/miR-146a tumor tissues by TUNEL assay. All data were represented as mean value ± SD from 3 independent experiments. **p < 0.01, ***p < 0.001

To further confirm the effects of miR-146a on the chemosensitivity of A549/DDP cells in vivo. Both A549 and A549/DDP cells stably transfected with miR-146a or NC were subcutaneously injected into nude mice, followed by treatment with DDP. As shown in Fig. 8a, the tumors formed from A549/DDP cells stably transfected with miR-146a grew significantly slowly than those from empty vector transfected cells, which indicated that up-regulation of miR-146a inhibited tumor growth. RT-qPCR analysis found miR-146a was significantly elevated and in tumor tissues formed from miR-146a-transfected A549//DDP cells than those from controls (Fig. 8b, *p* < 0.01). Moreover, up-regulation of miR-146a led to a significant decrease of CCNJ in tumor tissues, as determined by RT-qPCR, western blot and IHC, respectively (Fig. 8c, d and e). Furthermore, drug-resistance-associated proteins (P-gp, MRP1 and LRP) were down-regulated in A549/DDP/miR-146a compared with NC group and apoptosis-related protein (cleavedcaspase-3) were up-regulated by Western blot (Fig. 8d). TUNEL staining revealed increased apoptotic cells in tumors generated from miR-146a groups compared with the NC group (Fig. 8f). These results further demonstrated miR-146a might play an important role in increasing the chemosensitivity of A549/DDP cells to DDP by targeting CCNJ in vivo.

## Discussion

Cisplatin is the most widely used chemotherapy drugs for the treatment of lung cancer and other tumors [37]. Approximately 1,590,000 lung cancer patients succumb to the disease every year, 61% of which are primary drug resistance and 33% have an acquired drug resistance. Thus, an intense research underlying chemoresistance should be conducted to further establish better therapeutic approaches.

Currently, more and more studies focus on the research on oncogenes [38], epigenetic dysregulation [39] and abnormal expression of key genes (especially miRNAs) [10, 40] in drug resistance. Moreover, a series of miRNAs have been proposed as DDP resistance-associated genes according to miRNA microarray or RT-qPCR array profiling between A549 and A549/DDP cells [19, 41].

Most of studies have reported that miR-146a acts as an oncogene involved in tumor genesis and development [42], but other studies have demonstrated miR-146a functions as a tumor suppressor [43, 44]. Wu, C. et al. found that serum levels of miR-146a were potential biomarkers for the prediction of survival and response to chemotherapy in NSCLC [45]. Recently, direct targeting of EGFR by miR-146a was reported in castration-prostate cancer and HCC cells, leading to significant inhibition cell growth, colony formation, and migration in vitro [46, 47]. These evidences suggest that miR-146a plays an important role in the development and progression of cancer.

To our best knowledge, the role of miR-146a expression in DDP-resistent NSCLC has not been well documented. Interestingly, we found that miR-146a was down-regulated approximately 2-fold in A549/DDP cells, which attracts us to deeply explore the role of miR-146a in DDP-resistant NSCLC cells in vitro and vivo. Functional analysis indicated that miR-146a overexpression could sensitize NSCLC/DDP cells to DDP both in vitro and vivo by inducing G0/G1 phase arrest, inhibiting cell motility, and enhancing cell apoptosis. Therefore, we proposed that miR-146a might act as a chemosensitivity restorer to DDP in human NSCLC cells.

Further silico analysis showed that CCNJ was the target gene of miR-146a. The relevance between miR-146a and CCNJ was subsequently validated by luciferase reporter gene assay. As we know, CCNJ is a member of cyclin family protein that controls cell mitosis involved in oncogenesis and embryogenesis by forming CDK2/CCNJ complexes [48, 49]. Venturutti, L. et al. further suggest that the inhibition of CCNJ could repair the proliferation of breast carcinoma (BC) and gastric carcinoma (GC) cells in vitro and promote chemosensitive to trastuzumab and lapatinib in preclinical BC model [50]. A previous study also indicated that CCNJ could be a novel prognostic marker of HCC and acute leukemia (ALM) [51, 52]. To further investigate the effect of CCNJ in DDP-resistent NSCLC, both A549/DDP and SPC-A1/DPP cells were transfected with siCCNJ and then treated with DDP. As expected, we found that knockdown of CCNJ increased cell sensitivity to DDP by inducing cell cycle arrest and cell apoptosis. Notably, we found downregulation of CCNJ could enhance the gains of the sensitivity to DDP in miR-146a-overexpressing A549/DDP and SPC-A1/DDP cells.

There are several cell signaling molecules and pathways involved in drug resistance, including the ABC transporter subfamily B member 1 (ABCB1/MDR1/P-gp) [53, 54], ABC transporter subfamily C member 1 (ABCC1/MRP-1) [54], and lung resistance-related protein (LRP) [55]. Consistant with these reports, our results further demonstrated that miR-146a could downregulate drug-resistance-associated proteins (P-gp, MRP1 and LRP) and upregulate the expression of cleaved caspase-3 in vitro and vivo. However, we found P53, as a tumor suppressor, presented slightly downregulated in NSCLC/DDP cells after treated with miR-146a or siCCNJ, but no obvious change in tumor formed from A549/DDP cells stably transfected with miR-146a. These might be ascribed to cell growth status and different types of cells. Collectively, our results revealed that miR-146a overexpression could obviously increase the chemosensitivity of NSCLC/DDP cells to DDP by downregulating drug-resistance-associated proteins.

## Conclusions

In summary, we firstly reported miR-146a was downregulated was in DDP-resistant human NSCLC cells (A549/DDP and SPC-A1/DDP) compared with the sensitive parental cell line A549 and SPC-A1, and further demonstrated that miR-146a might act as a chemosensitivity restorer to DDP in human NSCLC cells by targeting CCNJ and downregulating P-gp, MRP1 and LRP. Based on these evidences, our experimental data may provide a novel therapeutic application of targeting the miR-146a/CCNJ interaction to treat DDP-based regimens-resistant NSCLC.

## Abbreviations

ABCB1: ABC transporter subfamily B member 1
ABCC1: ABC transporter subfamily C member 1
DDP: cis-diamminedichloroplatinum
HRP: Horseradish-peroxidase
LRP: Lung resistance-related protein
NSCLC: Non-small cell lung cancer
RT-qRCR: Real time quantitative PCR
SiRNA: Small interfering RNA

## References

[1] Ferlay J, Soerjomataram I, Dikshit R, Eser S, Mathers C, Rebelo M (2015). Cancer incidence and mortality worldwide: sources, methods and major patterns in GLOBOCAN 2012. Int J Cancer.

[2] Shen DW, Pouliot LM, Hall MD, Gottesman MM (2012). Cisplatin resistance: a cellular self-defense mechanism resulting from multiple epigenetic and genetic changes. Pharmacol Rev.

[3] Judson I, Kelland LR (2000). New developments and approaches in the platinum arena. Drugs.

[4] Reed JC (1999). Mechanisms of apoptosis avoidance in cancer. Curr Opin Oncol.

[5] Oliver TG, Mercer KL, Sayles LC, Burke JR, Mendus D, Lovejoy KS (2010). Chronic cisplatin treatment promotes enhanced damage repair and tumor progression in a mouse model of lung cancer. Genes Dev.

[6] Rosell R, Lord RV, Taron M, Reguart N (2002). DNA repair and cisplatin resistance in non-small-cell lung cancer. Lung Cancer.

[7] Safaei R, Howell SB (2005). Copper transporters regulate the cellular pharmacology and sensitivity to Pt drugs. Crit Rev Oncol Hematol.

[8] Materon EM, Jimmy Huang PJ, Wong A, Pupim Ferreira AA, Sotomayor Mdel P, Liu J (2014). Glutathione-s-transferase modified electrodes for detecting anticancer drugs. Biosens Bioelectron.

[9] Kirschner K, Melton DW (2010). Multiple roles of the ERCC1-XPF endonuclease in DNA repair and resistance to anticancer drugs. Anticancer Res.

[10] Magee P, Shi L, Garofalo M (2015). Role of microRNAs in chemoresistance. Ann Transl Med.

[11] Ambros V (2004). The functions of animal microRNAs. Nature.

[12] Bartel DP (2004). MicroRNAs: genomics, biogenesis, mechanism, and function. Cell.

[13] Cheng AM, Byrom MW, Shelton J, Ford LP (2005). Antisense inhibition of human miRNAs and indications for an involvement of miRNA in cell growth and apoptosis. Nucleic Acids Res.

[14] Krol J, Loedige I, Filipowicz W (2010). The widespread regulation of microRNA biogenesis, function and decay. Nat Rev Genet.

[15] Xu P, Guo M, Hay BA (2004). MicroRNAs and the regulation of cell death. Trends Genet.

[16] Moitra K, Im K, Limpert K, Borsa A, Sawitzke J, Robey R (2012). Differential gene and microRNA expression between etoposide resistant and etoposide sensitive MCF7 breast cancer cell lines. PLoS One.

[17] Naidu S, Garofalo M (2015). microRNAs: An Emerging Paradigm in Lung Cancer Chemoresistance. Front Med (Lausanne).

[18] Wu L, Pu X, Wang Q, Cao J, Xu F, Xu LI (2016). miR-96 induces cisplatin chemoresistance in non-small cell lung cancer cells by downregulating SAMD9. Oncol Lett.

[19] Li W, Wang W, Ding M, Zheng X, Ma S, Wang X (2016). MiR-1244 sensitizes the resistance of non-small cell lung cancer A549 cell to cisplatin. Cancer Cell Int.

[20] Chen X, Jiang Y, Huang Z, Li D, Chen X, Cao M (2016). miRNA-378 reverses chemoresistance to cisplatin in lung adenocarcinoma cells by targeting secreted clusterin. Sci Rep.

[21] Yang Z, Fang S, Di Y, Ying W, Tan Y, Gu W (2015). Modulation of NF-kappaB/miR-21/PTEN pathway sensitizes non-small cell lung cancer to cisplatin. PLoS One.

[22] Sui C, Meng F, Li Y, Jiang Y (2015). miR-148b reverses cisplatin-resistance in non-small cell cancer cells via negatively regulating DNA (cytosine-5)-methyltransferase 1(DNMT1) expression. J Transl Med.

[23] Cao J, He Y, Liu HQ, Wang SB, Zhao BC, Cheng YS (2015). MicroRNA 192 regulates chemo-resistance of lung adenocarcinoma for gemcitabine and cisplatin combined therapy by targeting Bcl-2. Int J Clin Exp Med.

[24] Zhao Z, Liu J, Wang C, Wang Y, Jiang Y, Guo M (2014). MicroRNA-25 regulates small cell lung cancer cell development and cell cycle through cyclin E2. Int J Clin Exp Pathol.

[25] Shi Y, Lu J, Zhou J, Tan X, He Y, Ding J (2014). Long non-coding RNA Loc554202 regulates proliferation and migration in breast cancer cells. Biochem Biophys Res Commun.

[26] Ning FL, Wang F, Li ML, Yu ZS, Hao YZ, Chen SS (2014). MicroRNA-182 modulates chemosensitivity of human non-small cell lung cancer to cisplatin by targeting PDCD4. Diagn Pathol.

[27] Li J, Wang Y, Song Y, Fu Z, Yu W (2014). miR-27a regulates cisplatin resistance and metastasis by targeting RKIP in human lung adenocarcinoma cells. Mol Cancer.

[28] Chen DQ, Pan BZ, Huang JY, Zhang K, Cui SY, De W (2014). HDAC 1/4-mediated silencing of microRNA-200b promotes chemoresistance in human lung adenocarcinoma cells. Oncotarget.

[29] Feng B, Wang R, Song HZ, Chen LB (2012). MicroRNA-200b reverses chemoresistance of docetaxel-resistant human lung adenocarcinoma cells by targeting E2F3. Cancer.

[30] Feng B, Wang R, Chen LB (2012). MiR-100 resensitizes docetaxel-resistant human lung adenocarcinoma cells (SPC-A1) to docetaxel by targeting Plk1. Cancer Lett.

[31] Wang X, Tang S, Le SY, Lu R, Rader JS, Meyers C, et al. Aberrant Expression of Oncogenic and Tumor-Suppressive MicroRNAs in Cervical Cancer Is Required for Cancer Cell Growth. PLoS One. 2008;3(7):e2557. 10.1371/journal.pone.0002557 PMC2438475 18596939

[32] He H, Jazdzewski K, Li W, Liyanarachchi S, Nagy R, Volinia S (2005). The role of microRNA genes in papillary thyroid carcinoma. Proc Natl Acad Sci U S A.

[33] Pacifico F, Crescenzi E, Mellone S, Iannetti A, Porrino N, Liguoro D (2010). Nuclear factor-{kappa}B contributes to anaplastic thyroid carcinomas through up-regulation of miR-146a. J Clin Endocrinol Metabol.

[34] Bhaumik D, Scott GK, Schokrpur S, Patil CK, Campisi J, Benz CC (2008). Expression of microRNA-146 suppresses NF-kappaB activity with reduction of metastatic potential in breast cancer cells. Oncogene.

[35] Li Y, Vandenboom TG, Wang Z, Kong D, Ali S, Philip PA (2010). miR-146a suppresses invasion of pancreatic cancer cells. Cancer Res.

[36] Wang G, Zhang M, Li Y, Zhou J, Chen L. Studying the effect of downregulating autophagy-related gene LC3 on TLR3 apoptotic pathway mediated by dsRNA in hepatocellular carcinoma cells. Cancer Res Treat. 2017;49(1):230–245. 10.4143/crt.2015.506 PMC5266382 27338037

[37] Chang A (2011). Chemotherapy, chemoresistance and the changing treatment landscape for NSCLC. Lung Cancer.

[38] Chang JC, Wooten EC, Tsimelzon A, Hilsenbeck SG, Gutierrez MC, Tham YL (2005). Patterns of resistance and incomplete response to docetaxel by gene expression profiling in breast cancer patients. J Clin Oncol.

[39] Kastl L, Brown I, Schofield AC (2010). Altered DNA methylation is associated with docetaxel resistance in human breast cancer cells. Int J Oncol.

[40] Kim ES (2016). Chemotherapy resistance in lung cancer. Adv Exp Med Biol.

[41] Wang Q, Zhong M, Liu W, Li J, Huang J, Zheng L (2011). Alterations of microRNAs in cisplatin-resistant human non-small cell lung cancer cells (A549/DDP). Exp Lung Res.

[42] Li L, Chen XP, Li YJ (2010). MicroRNA-146a and human disease. Scand J Immunol.

[43] Sun Q, Zhao X, Liu X, Wang Y, Huang J, Jiang B (2014). miR-146a functions as a tumor suppressor in prostate cancer by targeting Rac1. Prostate.

[44] Li H, Xie S, Liu M, Chen Z, Liu X, Wang L (2014). The clinical significance of downregulation of mir-124-3p, mir-146a-5p, mir-155-5p and mir-335-5p in gastric cancer tumorigenesis. Int J Oncol.

[45] Wu C, Cao Y, He Z, He J, Hu C, Duan H (2014). Serum levels of miR-19b and miR-146a as prognostic biomarkers for non-small cell lung cancer. Tohoku J Exp Med.

[46] Huang S, He R, Rong M, Dang Y, Chen G (2014). Synergistic effect of MiR-146a mimic and cetuximab on hepatocellular carcinoma cells. Biomed Res Int.

[47] Xu B, Wang N, Wang X, Tong N, Shao N, Tao J (2012). MiR-146a suppresses tumor growth and progression by targeting EGFR pathway and in a p-ERK-dependent manner in castration-resistant prostate cancer. Prostate.

[48] Althoff F, Viktorinova I, Kastl J, Lehner CF (2009). Drosophila Cyclin J is a mitotically stable Cdk1 partner without essential functions. Dev Biol.

[49] Kolonin MG, Finley RL (2000). A role for cyclin J in the rapid nuclear division cycles of early Drosophila embryogenesis. Dev Biol.

[50] Venturutti L, Cordo Russo RI, Rivas MA, Mercogliano MF, Izzo F, Oakley RH, et al. MiR-16 mediates trastuzumab and lapatinib response in ErbB-2-positive breast and gastric cancer via its novel targets CCNJ and FUBP1. Oncogene. 2016;35(48):6189–6202. 10.1038/onc.2016.151 PMC5832962 27157613

[51] Takano N, Hishida M, Inokawa Y, Hayashi M, Kanda M, Nishikawa Y (2015). CCNJ detected by triple combination array analysis as a tumor-related gene of hepatocellular carcinoma. Int J Oncol.

[52] Harvey RC, Mullighan CG, Wang X, Dobbin KK, Davidson GS, Bedrick EJ (2010). Identification of novel cluster groups in pediatric high-risk B-precursor acute lymphoblastic leukemia with gene expression profiling: correlation with genome-wide DNA copy number alterations, clinical characteristics, and outcome. Blood.

[53] Zhou W, Wang J, Man WY, Zhang QW, Xu WG (2015). siRNA silencing EZH2 reverses cisplatin-resistance of human non-small cell lung and gastric cancer cells. Asian Pac J Cancer Prev.

[54] Li JH, Luo N, Zhong MZ, Xiao ZQ, Wang JX, Yao XY (2016). Inhibition of microRNA-196a might reverse cisplatin resistance of A549/DDP non-small-cell lung cancer cell line. Tumour Biol.

[55] Janikova M, Zizkova V, Skarda J, Kharaishvili G, Radova L, Kolar Z (2016). Prognostic significance of miR-23b in combination with P-gp, MRP and LRP/MVP expression in non-small cell lung cancer. Neoplasma.

